# Anthropometric and mechanical factors determining sprint in young soccer players: a brief report

**DOI:** 10.3389/fspor.2024.1480973

**Published:** 2024-10-24

**Authors:** Alejandro Bustamante-Garrido, Esteban Aedo-Muñoz, Ciro Brito, Danilo Silva-Esparza, Jorge Pérez-Contreras, Mikel Izquierdo-Redin, Hugo Cerda-Kohler

**Affiliations:** ^1^ Navarrabiomed, Hospitalario Universitario de Navarra (HUN), Universidad Pública de Navarra (UPNA), IdiSNA, Pamplona, Spain; ^2^ Escuela de Ciencias del Deporte y la Actividad Física, Facultad de Salud, Universidad Santo Tomás, Santiago, Chile; ^3^ Laboratorio de Biomecánica Deportiva, Unidad de Ciencias Aplicadas al Deporte, Instituto Nacional de Deportes, Ministerio del Deporte de Chile, Santiago, Chile; ^4^ Escuela de Ciencias de la Actividad Física, el Deporte y la Salud, Facultad de Ciencias Médicas, Universidad de Santiago de Chile, Santiago, Chile; ^5^ Postgraduate Program of Physical Education, Federal University of Juiz de Fora, Governador Valadares, Belo Horizonte, Brazil; ^6^ Área física de fútbol formativo de Universidad Católica de Chile – Cruzados SDAP, Santiago, Chile; ^7^ Escuela de Educación, programa de Magíster en Evaluación y Planificación del Entrenamiento Deportivo, Universidad Viña del mar, Viña del Mar, Chile; ^8^ Escuela de Doctorado en Investigación Aplicada a las Ciencias Sanitarias, Universidad de las Palmas de Gran Canaria, Las Palmas de Gran Canaria, Spain; ^9^ Unidad de Fisiología del Ejercicio, Centro de Innovación, Clínica MEDS, Santiago, Chile; ^10^ Departamento de Educación Física, Deportes y Recreación, Facultad de Artes y Educación Física, Universidad Metropolitana de Ciencias de la Educación, Santiago, Chile

**Keywords:** athletic performance, anthropometry, force-velocity profile, motion analysis, soccer

## Abstract

Sprint performance is a critical factor in soccer. While previous studies have extensively explored the biomechanical, physiological, and metabolic determinants of sprinting, the impact of anthropometric variables in team sports contexts, especially soccer, remains underexplored. This study aims to investigate the influence of anthropometric and mechanical variables on sprint performance in young soccer players. Fifty-eight young soccer players were evaluated in anthropometry and a 30-meter (m) sprint using radar technology. Split times in 5, 15, and 30 m were determined, in addition to the assessment of the force-velocity profile proposed by Morin and Samozino. Results: Key anthropometric variables associated with improved sprint performance included lower-limb muscle mass at distances 5 and 15 m (*R*^2^ = 0.08 and *R*^2^ = 0.09, respectively, both with small effects). Additionally, body composition, particularly a lower % body fat, was crucial across all sprint distances (ES: large). Among the mechanical variables, max power (*R*^2^ = 0.997, ES: large) and maximum velocity (*R*^2^ = 0.553, ES: large) are the mechanical variables that were most strongly associated with sprint performance over distances greater than 30 m. Soccer coaches, athletic trainers, and strength and conditioning specialists working with young athletes can apply the findings of this study to their training programming.

## 1 Introduction

Successful soccer performance is influenced by various modifiable physical, physiological, biomechanical, and tactical factors (1). During a soccer match, players must repeatedly alternate between low- and high-intensity efforts, covering large distances with frequent multidirectional movements. These movements often include sprints, jumps, changes of direction, lateral displacements, and quick decelerations (2). Sprinting actions, in particular, are crucial for soccer performance. On average, professional soccer players perform between 27 and 35 sprints per match (3), each lasting 2–4 s (4). These sprints are decisive for critical moments in the game, such as counter-attacks, pressing, and positional advantages (5), and can significantly affect match outcomes.

**Figure 1:**
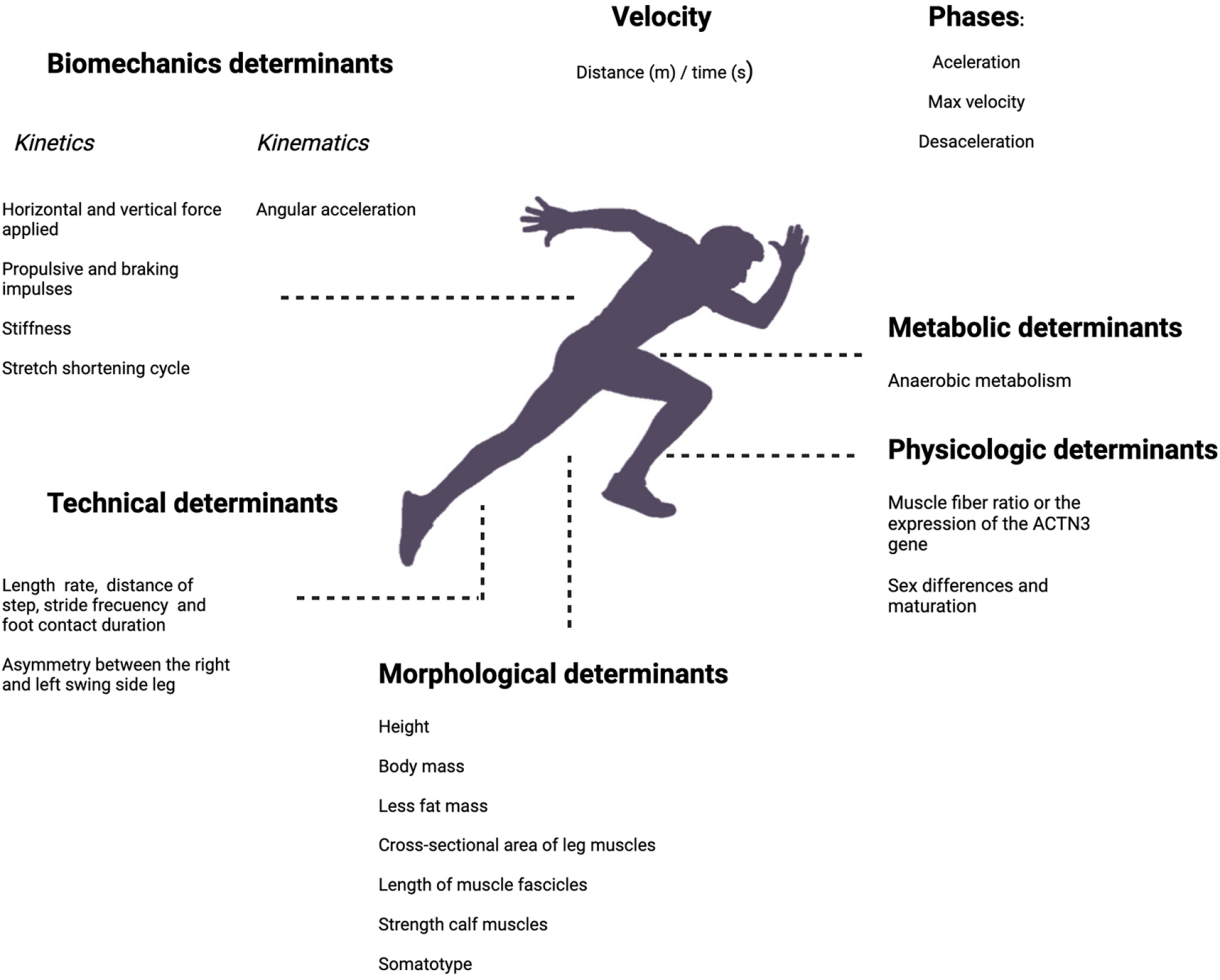
Kinetics: horizontal and vertical force applied (6–9, 16, 17); propulsive and braking impulses (10, 18–20); stiffness (21, 22); stretch-shortening cycle (21, 22), kinematics: angular acceleration (17, 23, 24), technical determinants: step length, rate, and distance, stride frequency and foot contact duration (18); timing of the contralateral arm and leg swing, asymmetry between the right and left swing side leg (25), morphological determinants; height (13), body mass (11, 12), less fat mass (26), cross-sectional area of leg muscles (27–30), length of muscle fascicles (31), strength calf muscles (32), somatotype (33); physiologic determinants: muscle fiber ratio or the expression of the ACTN3 gene (14, 34); sex differences and maturation (13, 35–37), anaerobic metabolism (15).

Accelerating and reaching top speed depends on a complex interplay of physiological, biomechanical, metabolic, and morphological factors (6). Strength, power, and impulse are key biomechanical contributors (6–10). Morphological factors such as body stature and muscle mass influence a player's sprinting capacity (11–13). Additionally, factors like muscle fiber composition (14) and anaerobic capacity (15) affect an athlete's ability to perform repeated high-intensity efforts. While genetic traits, sex, and age inevitably shape physical capacities, combining these unmodifiable factors with trainable attributes ultimately determines performance in soccer. These and other factors encompass a range of variables contributing to sprint performance, as schematically represented in Figure 1. However, research on the impact of morphological factors on speed and acceleration performance still needs to be conducted.

Regarding anthropometric variables affecting sprint performance, most studies have focused on track and field sprinters rather than team sports athletes (see Figure 1 for references related to morphological determinants). A few studies have identified the anthropometric variables that affect sprinting in team sports such as soccer. For example, body mass (BM), body mass index (BMI), and height (13, 38) have been variables associated with sprint performance. This presents a significant gap in the literature, particularly for soccer, in which the specific demands and context differ markedly from individual sprint events. Our study addressed this gap by examining anthropometric variables influencing young soccer players’ sprint performance. By doing so, we hope to provide insights that will inform training and development strategies tailored to the unique needs of soccer athletes.

## 2 Materials and methods

### 2.1 Experiment approach

The assessments were conducted on cadets from Chile's first-division professional soccer teams. These evaluations were part of the battery of tests performed by the staff, and the players signed informed consent and permission for their data to be published. The assessments were carried out following the Declaration of Helsinki standards.

### 2.2 Participants

Fifty-eight male cadet players (age: 16.05 + 1.46 years, height: 175.14 + 7.10 cm, body mass: 65.78 + 8.26 kg) participated in this study. Goalkeepers were discarded due to the specificity of their positions.

### 2.3 Anthropometric assessments

All athletes were evaluated in the morning after fasting for 12 h. The assessment of body mass was conducted using a Tanita precision scale, model Bc-601, with a resolution of 0.1 g. Height evaluation was performed using a SECA brand stadiometer with 1 mm precision. A Lufkin brand metal tape measure with an accuracy of 1 mm was employed to measure the body circumferences. Diameter measurements were obtained using a Rosskraft caliper, while skinfold thickness was assessed using a Harpenden caliper. The assessments were performed following the protocols established by ISAK (39) by a certified level II professional.

### 2.4 Speed-acceleration evaluation 30 m

The 30 m acceleration evaluation was performed on a natural grass soccer field with specific footwear for this surface type. For this, Stalker ATS II model radar (Applied Concepts, Dallas, TX, USA; accuracy + 1.61 km/h, sampling 46.9 Hz) was located on a tripod 10 m from the starting line and at the height of 1 m to align with the location of the center of mass (CM) of the subject (5). The athletes were instructed to initiate a 30 m maximal sprint starting from the standing starting position. Two attempts were performed with 5 min rest between them. Sprint performance metrics (split times at 0–5, 0–15, and 0–30 m) and mechanical outputs were analyzed based on the best time trial performance.

### 2.5 Horizontal force-velocity profile (FVP)

The speed-time data obtained via radar were processed using the Excel® spreadsheet developed by Morin and Samozino (40). This tool facilitates the computation of the maximum horizontal force exerted during the sprint (F0), maximum velocity (V0), and peak horizontal power output (Pmax). Furthermore, we determined the proportion of the total lower limb force applied horizontally to the ground (RF_max_) and the rate of decline in horizontal force with increasing velocity (DRF). This approach relies on the fundamental principles of motion to establish the force-velocity relationship utilizing athlete velocity and body mass (41). Radar validity for these purposes was confirmed through comparison with force platforms (absolute bias of 3%–7%) (42). In terms of reliability, the mean typical error remained small (CV ≤ 8.4%) across all kinetic and kinematic variables (43), confirming its utility as a field tool for assessing these parameters.

To model the net horizontal anteroposterior ground reaction force (FH) applied to the body's center of mass (CM) over time, we employed the following equation (43):

FH(t)=m*aH(t)+Faero(t)

here, *m* denotes the runner's body mass in kilograms, and Faero(*t*) represents the aerodynamic drag to be overcome during sprinting, proportional to the square of the air velocity relative to the runner:

Faero(t)=k*(vH(t)−vw)2

where vw is the wind velocity (if applicable), and *k* is the runner's aerodynamic friction coefficient. In the vertical direction, during the acceleration phase, the runner's body CM ascended from the initial to an upright running posture and remained constant between the complete strides. Thus, by utilizing fundamental dynamics laws in the vertical plane, the mean net vertical ground reaction forces (FV) applied to the body CM during each complete stride can be modeled over time as equal to the body weight:

FV(t)=m*g

here, “g” denotes the gravitational acceleration (9.81 m/s^2^).

The mechanical effectiveness of force application during running can be quantified in each support phase or stride by the ratio (RF in%) of FH to the corresponding total resultant ground reaction forces (FRes, in *N*) and throughout the entire acceleration phase by the slope of the linear decrease in RF with increasing velocity (DRF, in %/s/m). Since the starting block phase (push-off and subsequent airborne time) lasts between 0.5 and 0.6 s (44, 45), occurring for an average duration of ∼0.3 s, RF and DRF values can be computed from FH and FV values modeled for *t* > 0.3 s.

## 3 Statical analysis

Stepwise linear regression analysis was performed to identify the determinants of sprint performance. Before these analyses, a collinearity diagnostic procedure was implemented to reduce possible multicollinearity problems among predictor variables. Three linear regressions were performed: anthropometric variables vs. time, mechanical variables vs. time, and a combination of anthropometric and mechanical variables vs. time. The latter was performed to determine the relative contribution of each factor. The effect size (ES) for multiple linear regressions was calculated using Cohen's *f*^2^ (46). The following threshold values for ES reported as *f*^2^ were employed: ≥0.02 as small, ≥0.15 as medium, and ≥0.35 as large (47). Stata (Release 18. College Station, TX: StataCorp LLC) software was used for these analyses.

## 4 Results

The results of our study indicate that the mean and standard deviation times to cover 5, 15 and 30 m were: 1.42 ± 0.10, 2.83 ± 0.12 and 4.66 ± 0.20 s respectively. The variables derived from the FVP were F0 (N/kg) 7.07 ± 0.82, V0 (m/s^2^) 9.18 ± 0.54 and Pmax (W/kg) 16.23 ± 2.19. The applied linear regressions showed that body mass index (BMI, 21.39 ± 1.85), % muscle mass (%MM, 50.20 ± 5.82), % body fat (%BF, 21.07 ± 3.30), maximal hip circumference (MHC, 94.27 ± 4.89) and TCC (thigh circumference corrected, 55.86 ± 4.53) are the anthropometric variables that affect the sprint.

### 4.1 Anthropometric variables determining sprint

When examining the anthropometric variables influencing 5 m sprint times, BMI and TCC were identified as the main factors. The stepwise linear regression model explained 8% of the variability in 5 m sprint times (*R*^2^ = 0.08), reaching statistical significance (*p* = 0.0384) with an *F*-value of 3.46. The model's RMSE is 0.09523, indicating an acceptable fit. Analysis of anthropometric variables for the 15 m sprint highlighted MHC as a significant determinant. The stepwise linear regression model accounted for 9% of the variability in the 15 m sprint times (*R*^2^ = 0.09), with statistical significance (*p* = 0.0128) and an *F*-value of 6.62, confirming the model's reliability. The model, with an *R*^2^ of 0.32, showed that BMI, % MM, and % BF collectively explained 32% of the variability in the 30 m sprint times (*p* < 0.001, *F* = 10.08). Table 1 presents the results of the stepwise linear regression analyses’ results examining anthropometric variables’ influence on the 5-, 15, and 30 m sprint times. For each distance, the table displays each predictor variable's non-standardized coefficient (*B*), standard error, *t*-value, *p*-value, 95% confidence interval, *f*^2^ and ES.

**Table 1:** Anthropometric variables that determine sprints.

Variable	Coefficient (*B*)	Standar error	*t*-value	*p*-value	95% expected for *B*		*R* ^2^	Cohen's *f*^2^	ES
					Upper limit	Lower limit			
5 m time									
Intercept	1.62320	0.2020348	8.03	0.000	1.218403	2.028176	0.08	0.09	Small
BM	0.0074796	0.0028567	2.62	0.011	0.0017545	0.0132046			
TCC	−0.0138922	0.006688	−2.08	0.042	−0.00272952	−0.0004893			
15 m time									
Intercept	3.599001	0.2998898	12.00	0.000	2.99825	4.199753	0.09	0.10	Small
MHC	−0.0081717	0.0031768	−2.57	0.013	−0.0145357	−0.0018077			
30 m time									
Intercept	3.410726	0.6823492	5.00	0.000	2.042698	4.778753	0.32	0.47	Large
BMI	−0.311523	0.0127759	−0.244	0.018	−0.567665	−0.005382			
% MM	0.0190374	0.0066525	2.86	0.006	0.0056999	0.032375			
% BF	0.0454014	0.0123178	3.69	0.001	0.0207057	0.0700971			

BM, body; TTC, thigh circumference corrected; MHC, maximum hip circumference; % MM, muscle mass percentage; % BF, body fat percentage.

### 4.2 Mechanical variables determining sprint

Stepwise linear regression identified the relative Pmax as the primary mechanical variable that influenced the time to run 5 m. The model explained 16.8% of the variability in 5 m sprint times (*R*^2^ = 0.168). This coefficient suggests that higher values of relative Pmax are associated with shorter 5 m sprint times. For the 15 m distance, relative Pmax was also identified as the primary mechanical variable explaining sprint time (97.57%; *R*^2^ = 0.9757). The analysis identified V0 as the primary mechanical variable influencing the time to run at 30 m. The model explains 55.34% of the variability (*R*^2^ = 0.5534). Table 2 presents the results of stepwise linear regression.

**Table 2:** Mechanical variables that determine sprints.

Variable	Coefficient (*B*)	Standar error	*t*-value	*p*-value	95% expected for *B*		*R* ^2^	Cohen's *f*^2^	ES
					Upper limit	Lower limit			
5 m time									
Intercept	1.737302	0.0897863	19.35	0.000	1.557439	1.917166	0.168	0.20	Medium
Pmax	−0.0194284	0.005485	−3.54	0.001	−0.0304152	−0.0084406			
15 m time									
Intercept	3.731035	0.0190149	196.22	0.000	3.692944	3.769127	0.975	32.3	Large
Pmax	−0.0556175	0.0011616	−47.88	0.000	−0.0579445	−0.0532905			
30 m time									
Intercept	7.065933	0.2852099	24.77	0.000	6.494589	7.637277	0.553	1.24	Large
V0	−0.262549	0.031024	−0.46	0.000	−0.3246975	−0.2004004			

Pmax, maximal horizontal power; V0, maximal theoretical velocity.

### 4.3 Mechanical and anthropometric variables determining sprint

Analysis of the mechanical and anthropometric variables affecting 5 m sprint times revealed significant results. The model explained 36.01% (*R*^2^ = 0.3601) of the variance in 5 m sprint times (*p* < 0.0001, *F* = 11.69). The coefficients indicated that a higher Pmax was associated with shorter 5 m sprint times, whereas an increase in MCH was related to longer sprint times. For 15 m, no anthropometric variable was added, leaving only Pmax as the most determining variable of this distance. Our model explained 59.75% of the variability in the 30 m sprint times (*R*^2^ = 0.5975, *p* < 0.0001, *F* = 29.20). The coefficients indicate that a higher V0 and a larger TCC are associated with reduced sprint times, whereas an increased MHC is linked to longer sprint times. Table 3 presents the results of stepwise linear regression for 30 m.

**Table 3:** Anthropometric and mechanicals variables that determine sprints.

Variable	Coefficient (*B*)	Standar error	*t*-value	*p*-value	95% expected for *B*		*R* ^2^	Cohen's *f*^2^	ES
					Upper limit	Lower limit			
5 m time									
Intercept	1.245035	0.2224322	5.6	0.000	0.7992692	1.690802	0.360	0.56	Large
Pmax	−0.0237657	0.005567	−4.27	0.000	−0.0349222	−0.0126093			
MHC	0.0059681	0.005567	2.40	0.020	0.000986	0.109502			
30 m time									
Intercept	6.632695	0.3762495	17.63	0.000	5.87836	7.38703	0.597	1.48	Large
MHC	0.0182737	0.0073609	2.50	0.015	0.0036243	0.0329232			
TCC	−0.0296856	0.0104207	−2.85	0.006	−0.0505779	−0.0087933			
V0	−0.2416436	0.0341626	−7.07	0.000	−0.3101354	−0.1731517			

Pmax, maximal horizontal power; MHC, maximum hip circumference; TTC, thigh circumference corrected; V0, maximal theoretical velocity.

## 5 Discussion

Our study aimed to identify the key anthropometric and mechanical variables influencing sprint performance. Understanding these factors is crucial because the ability to execute such high-speed actions significantly enhances the likelihood of success in both offensive and defensive football actions.

### 5.1 Anthropometric variables related to sprint performance

Regarding anthropometric variables related to sprinting, our results align with those of previous studies (48–50), where higher BMI values were negatively associated with performance in the 5 and 30-meter sprints (small and large ES, respectively). Thus, the most critical indicator is body composition, where lower-limb MM is associated with better sprint performance (51). Our results support this: TCC and MHC are anthropometric variables associated with better performance at 5 and 15 m (ES: small), respectively. Chelly et al. (52) report significant correlations between thigh muscle volume and 5 m sprint performance in young soccer players (*r* = 0.43, *p* = 0.05), and Tottori et al. (53) identified the cross-sectional area of the gluteus maximus as a predictive variable in 100-m performance in adults sprinters. Similar to other studies (54–56), our results showed a negative association between% BF and short-distance sprint times and % MM. This result contradicts our hypothesis, which can be explained by the contribution of MM to the total BM. These results provide valuable insights into MM gain, specifically in the lower limbs, reduction of %BF, and optimal BM to enhance sprint performance in football players.

### 5.2 Mechanical variables related to sprinting

Our results indicate that the relative Pmax is the variable that best explains the time to cover 5 and 15 m (medium and large ES). At the same time, V0 is the mechanical variable that has the most significant influence on the 30-meter sprint time (ES: large). These findings are consistent with those of Haugen et al. (57), who found an almost perfect inverse correlation (*r* = 1 ± 0.01) between the time to cover short distances (10 m) and relative Pmax. They also observed that the V0 variable correlated more significantly as distance increased. Samozino et al. (58) emphasized that the Pmax and FV profile of each athlete were the most significant variables in short-distance sprints (>30 m). Other studies have reported that F0 and V0 are the variables that contribute most to the time over short distances (59, 60). While these studies may seem contradictory, we must clarify that mechanical power is the product of applied force and velocity (61). This means that Pmax is a composite variable that reflects the capacity to generate force and achieve a high velocity. Therefore, the results were similar at first glance. Pmax integrates the ability to effectively apply force during the initial acceleration and the capacity to maintain a high maximum velocity over longer distances. The results suggest that optimizing Pmax can improve the initial acceleration and achieve higher maximum speeds.

### 5.3 Anthropometric and mechanical variables that determine times over short distances

An analysis that considered anthropometric and mechanical variables was conducted to determine their relationship with sprint performance. Our results enhanced the accuracy over distances of 5 and 30 m. Pmax, V0, and TCC negatively affected the time to cover 5 m (higher values, shorter times; ES: large). The impact of Pmax on short distances has been discussed in the previous section. For 30 m, V0 and TCC contribute to reducing time (ES: large), while MHC slightly increases it. The latter may seem contradictory, as this variable has been shown to increase V0. Studies have highlighted the inconsistency in the relationship between sprint performance and hip extensors (62), emphasizing that they relate to the sprint phase (acceleration, maximum velocity, and deceleration). According to our results, MHC is an essential variable in the initial meters of the sprint (>15 m) but not in longer sprints.

## 6 Practical applications and limitations

The results provide valuable information for trainers, and strength and conditioning specialists working with young athletes as they can apply this study's findings directly, by incorporating exercises that target lower-limb muscle development, improve body composition, and enhance mechanical power output, coaches can design programs specifically tailored to strengthen sprinting ability, which may enhance overall soccer performance and increase the likelihood of athletic success.

It is important to highlight that the biological age of the participants could not be identified, which could have provided additional information regarding their physical development and maturation. Additionally, the sample size was determined by convenience, as we included all athletes in the available category, limiting the generalizability of the results to a broader population.

## 7 Conclusion

This study investigated the relationship between anthropometric and mechanical variables and sprint performance among young soccer players. Our findings provide valuable insights into optimizing training programs to improve this population's sprinting ability. Key anthropometric variables associated with improved sprint performance included lower-limb MM at short distances (5 and 15 m). Additionally, body composition, particularly a lower % BF, was crucial across all sprint distances. Among the mechanical variables, Pmax and theoretical V0 were most strongly associated with sprint performance (<30 m), as well as with distances greater than 30 m. This provides valuable information for youth soccer coaches and physical trainers. A notable result from our research is the negative relationship between MCH and the 30 m time. Future research should further explore the relative contribution of perimeter during different phases of sprinting.

## Data Availability

The raw data supporting the conclusions of this article will be made available by the authors, without undue reservation.
